# Popular Dental Education

**Published:** 1885-07

**Authors:** J. M. Clyde


### Popular Dental Education.

BY J. M. CLYDE, D.D.S.

Read before the Kentucky State Dental Association, June, 1885.

MR. PRESIDENT:—To me has been assigned the following theme: "How shall we educate the people up to the ideas of modern dentistry?"

I would have preferred something else, but your executive committee has given me no choice in this matter, and presuming that the will of the committee is like the laws of the Medes and Persians, I deem it best to resign myself to fate and venture a few thoughts for consideration.

It is a Herculean task to interest the masses in the progress of science in any of its departments. In our special field it is not so difficult to say something as it is to render that something practical. Unless practical it is useless. It is easy to make suggestions as to what ought to be done, but as to the how—well, here it ends. What imports it that we discuss this and kindred propositions at our meetings, if when we return home we leave all behind us? What values it that we know what to teach and how, if we relapse into our former state of passivity and fail to live up to the measure of our knowledge?

We frame laws regulating the practice of dentistry in the State and petition the legislature to place them on the statute books, but who has the temerity to see them faithfully executed? Our laws are good but are not worth the paper on which they are printed, because no one assumes the responsibility of making them practical. What respect have mountebanks for such laws? The people will patronize them until they are taught to discriminate between honesty and humbuggery. It is just so, too, in the general practice of medicine; the greatest advertiser and pretender, if he has the requisite impudence to make bold and unscrupulous assertions, is sure to win. But this brings us to our question, how shall we educate the people that they may be protected against such imposition.

1. We suggest, first, that it be made the duty of the executive committee, or of any one whom this association may deem proper, to see that the laws appertaining to the practice of our profession in this State are not infringed. If every State Association would do this I believe it would have a salutary effect. "What is everybody's business is nobody's business." The principle involved in this aphorism is the reason for the existence of officers to execute the laws, whether State, national, or municipal. Without them all would be anarchy. Let it then be the special business of some one to see that our laws are respected, that no person practices dentistry in this State, even to the extraction of a tooth, except in conformity with law. Let no man degrade our profession. Let us have a registration law sufficiently broad and comprehensive to "turn the rascals out."

2. Let your light so shine before men that they seeing your good works may be induced to participate in its blessings. Many of us circumscribe our knowledge within the narrow limits of the hat-band. If we possess knowledge, let others feel our power. The more the people are enlightened the better it will be both for us and them. Call their attention to the office of the dental organs in the animal economy. Show their necessity to health and beauty, and the possibilities of their preservation. There are various ways in which this may be done. A word fitly spoken to our patients in the chair may often be like apples of gold in pictures of silver. But I opine the chief means for the accomplishment of this end is in the publication of tracts or small books on dental subjects. In these two or three only of the most prominent subjects should be treated, and no attempt made to exhaust any; otherwise we would only weary the reader and frustrate our object. Our aim should be to instruct others, not to display ourselves. All that is necessary to be said might be condensed into one little volume of less than an hour's reading. A few points made strong is the way to gain the attention of the public. The contents of the book might be as follows: Part first. The Temporary Teeth, their necessity and means of preservation. Part second. The Permanent Teeth, their necessity and means of preservation, and part third. Calareous Deposits on the Teeth, their effects and treatment.

It might be well to have an appendix giving an accurate description of the quack so as to guard the unwary.

3. Much might be done in the office to educate the people up to our standard. A few minutes occasionally employed in removing calcareous deposits from a tooth where it is, though perhaps unsuspected, causing considerable annoyance, and kindly directing attention to the general condition of the oral cavity, the damage already done to the teeth and gums by the concretion, its effects on the health, breath and general appearance, then carefully cleansing a few teeth and directing the patient to notice the difference in the aspect of the gums in a day or two, will often do more to educate the mind to appreciate the necessity of a clean mouth and a pure sweet breath than as many hours of laborious reading. Is your patient dyspeptic, or neurotic, or both, endeavor to show him that his sufferings may be very greatly relieved by having his mouth put in a condition so that he can masticate his food without commingling it with those poisonous products of inflammation and decomposition constantly oozing from around the necks of his teeth, the result of tartar and decay.

Again, do not be in haste to extract an aching tooth especially if it is an important one and there is a fair probability that it can be made comfortable. The forceps ought to be a dernier resort. Carefully remove all the debris from the cavity of decay. If the pulp is exposed or very nearly so, an excellent dressing well adapted to very many cases of this kind because quickly and easily manipulated is the following:

℞ Oxide of zinc and creasote q.s., to make a soft paste. Spread this gently over the pulp and sponge off with bibulous paper, etc., being careful to avoid pressure. Over this with equal care spread a soft paste of oxychloride of zinc, completely covering it. After this has set firmly, trim and fill the cavity as may be deemed best. If the pulp is but slightly congested, such an operation well performed, success is almost sure, and much will be done to impress our patient with the importance of the dental organs. If we do not value them sufficiently to venture such an operation, even though but poorly remunerated, what can we expect of others? We should always, however, guard against giving too great assurance of success, for there is always a possibility of failure. In making a prognosis several things must be considered, viz., the age of the patient, his general health, his habits, taints of vice, whether hereditary or acquired, the condition of the pulp, as well as the character of the operation. It is well in such cases to describe what has been done and direct the patient to return in a few weeks or months, that you may have an opportunity of reviewing and, if necessary, completing the work. All will not do this, but if only one out of ten returns to give glory, you will fair no worse than did the great physician of souls in the case of the lepers. The others may have talked about it to their friends " allee samee."

We sometimes have to operate on teeth abnormally sensitive. Patients having such teeth always make an unfavorable impression. Such teeth should be treated with the cements until restored to a normal condition. This hyperæsthetic state is frequently dependent on a similar condition of the general system, which may be relieved by proper tonics, judicious exercise, change of habitation, etc. The excavation of such teeth need not be more thorough than can be accomplished by the aid of anæsthetics without much pain. What if the operation has to be repeated several times if our patient only appreciates its necessity? In this way we may do much to remove false impressions as to the exceedingly painful nature of the process, graphically described as " worse than pulling."

4. An excellent plan to induce people to place a proper estimate on the dental organs, could it only be made universal, would be to charge an enormous fee for extraction except in cases of absolute necessity, and make artificial teeth cost "like thunder." But here is where the quacks get us. They extract free (?) of charge in order to have an opportunity of making a set of cheap teeth. Unless some method can be adopted to stop these extractions by wholesale, our practice will necessarily fall below the standard of our knowledge. As it is, we are compelled to do some things that do not comport well with our ideas of modern dentistry. Here is the chief enemy to our progress, and the more so because he is unscrupulous. Honesty is entirely foreign to his nature. His schemes and his practice are based on the ignorance and credulity of the people. His supreme and sole object is filthy lucre. Gas, which was intended to be a blessing to suffering humanity, he has turned into a curse. Let the masses be properly informed on this subject and this mutilator of human mouths and individuality of expression will be forced to find employment in some other calling where by the nature of circumstances he may be compelled to honesty. Why, if a veterinary surgeon in his treatment of fine cattle and horses should exhibit as little genuine integrity in his dealings as does the dental quack he would be constantly in court defending himself against the charge of mal-practice.

But how are the people to distinguish the quack from the regular practitioner? We will give some of his ear marks:

"Beautiful sets of teeth for $5." "Extraction free of charge when artificial teeth are ordered." "All work warranted to give entire satisfaction."

"Gold fillings for $1 and upward." "Fresh gas daily."

Besides these quotations from his advertisements in the daily papers, hand-bills, signs, etc., he frequently exhibits specimens of artificial teeth in his windows, on the street, at shows and exhibitions, some of them hung on hinges and worked by machinery so as to imitate the movement of the jaws and attract special attention to the fact (?) that he makes teeth (?) for $5.

Let the people know that no respectable dentist does anything of this sort so disgusting to truly educated and refined taste, and much will be done to elevate the popular mind in the right direction.

In attempting to work out these suggestions care is necessary lest we degrade ourselves. There should be no effort made at self-aggrandizement, neither should we blush to claim all honors due.

Again, we should not become impatient because others do not see and appreciate these things immediately. Science is not a meteoric flash; it is an ever-increasing incandescence. We must learn to labor and to wait. If we never enjoy the reward of our labors, we will leave a legacy to the coming generations.

